# Socioeconomic Impact on the Prevalence of Cardiovascular Risk Factors in Wallonia, Belgium: A Population-Based Study

**DOI:** 10.1155/2015/580849

**Published:** 2015-08-25

**Authors:** Sylvie Streel, Anne-Françoise Donneau, Axelle Hoge, Sven Majerus, Philippe Kolh, Jean-Paul Chapelle, Adelin Albert, Michèle Guillaume

**Affiliations:** ^1^Department of Public Health, University of Liège, 4000 Liège, Belgium; ^2^Department of Health Economics Information, University Hospital of Liège, 4000 Liège, Belgium; ^3^Department of Laboratory Medicine, University Hospital of Liège, 4000 Liège, Belgium

## Abstract

*Background*. Monitoring the epidemiology of cardiovascular risk factors (CRFs) and their determinants is important to develop appropriate recommendations to prevent cardiovascular diseases in specific risk groups. The NESCaV study was designed to collect standardized data to estimate the prevalence of CRFs in relation to socioeconomic parameters among the general adult population in the province of Liège, Wallonia, Belgium. *Methods*. A representative stratified random sample of 1017 subjects, aged 20–69 years, participated in the NESCaV study (2010–2012). A self-administered questionnaire, a clinical examination, and laboratory tests were performed on participants. CRFs included hypertension, dyslipidemia, global obesity, abdominal obesity, diabetes, current smoking, and physical inactivity. Covariates were education and subjective and objective socioeconomic levels. Data were analyzed by weighted logistic regression. *Results*. The prevalence of hypertension, abdominal obesity, global obesity, current smoking, and physical inactivity was higher in subjects with low education and who considered themselves “financially in need.” Living below poverty threshold also increased the risk of global and abdominal obesity, current smoking, and physical inactivity. *Conclusion*. The study shows that socioeconomic factors impact the prevalence of CRFs in the adult population of Wallonia. Current public health policies should be adjusted to reduce health inequalities in specific risk groups.

## 1. Introduction

Cardiovascular diseases (CVD) are the first cause of death worldwide [1–3]. In Europe, CVD are responsible for 47% of all deaths (52% in women and 42% in men) with significant differences in mortality rates between countries [1]. CVD also contribute substantially to morbidity. In European countries, CVD are responsible for 17% of all disability-adjusted life years (DALYs) lost, making it the second largest single cause after neuropsychiatric disorders [1]. The annual cost of these diseases is estimated to amount to €196 billion in the European Union (EU). Moreover, 54% of the costs are due to direct health care budget, 24% to productivity losses, and 22% to informal care of people with CVD [1].

In the last decades, CVD-related mortality has declined markedly in many European countries [1–5]. The decreasing trend has been attributed to changes in the prevention and control of cardiovascular risk factors (CRFs) such as lifestyle factors, especially tobacco, unhealthy diet habits, and physical inactivity, and by the use of more effective medical and surgical treatments [3]. Adequate changes in lifestyle-related risk factors may prevent over 75% of all CVD deaths according to the World Health Organization (WHO) [4].

The higher morbidity and mortality rates observed in some specific groups, however, may be explained by the higher prevalence of CRFs in these groups itself due to socioeconomic differences such as educational level, occupational class, or income level [6, 7]. Adapted CVD prevention remains a major challenge for eradicating, eliminating, or minimizing the burden of CVD on health systems and societies [3] and also for reducing health inequalities [7]. The bases of prevention are rooted in cardiovascular epidemiology and evidence-based medicine [4]. In this context, three neighboring regions, Grand-Duchy of Luxembourg, Wallonia in Belgium, and Lorraine in France, respectively, faced with the lack of comparable data on cardiovascular health in Europe, joined together to conduct the “Nutrition, Environment and Cardiovascular Health” (NESCaV) project. Its main goal was to build an epidemiological surveillance tool for collecting standardized data to establish baseline information on the prevalence of several potentially modifiable CRFs and, thus, generate some recommendations to promote efficiently cardiovascular health in the so-called Greater Region. The NESCaV study was designed under the auspices of the European interregional program “INTERREG IV A” and used a standard approach for the three participating regions [8]. For the Belgian part, it was conducted in the province of Liège (Wallonia) between May 2010 and March 2012 by the Department of Public Health of the University of Liège, in collaboration with the University Hospital (CHU) of Liège.

The present work aimed (1) to determine the prevalence of potentially preventable and modifiable CRFs including hypertension, dyslipidemia, global obesity, abdominal obesity, diabetes, current smoking, and physical inactivity, among the general adult population of Wallonia, and (2) to investigate the potential impact of socioeconomic factors on the CRFs.

## 2. Methods

### 2.1. Sampling Design

A representative stratified random sample of subjects aged 20–69 years of the province of Liège was drawn from the Belgian national register of residents. Stratification was made by gender, age, and district of residence. Pregnant women and people living in institutions were excluded. A power calculation showed that a sample size of at least 1000 subjects was needed to estimate the prevalence of risk factors with a statistical precision of at least 2% [8]. A total of 1017 subjects eventually participated in the study. The study design and information collected were approved by the Ethics Committee of the Faculty of Medicine of the University of Liège (B70720097541).

### 2.2. Method of Recruitment

To solicit selected subjects to participate in the NESCaV project, an official letter was sent by the investigators to explain the study objectives, relevance to public health policies, ways of participation, tests to be performed, and participant's rights. Attached was another letter addressed to the family physician to inform and invite him/her to encourage participation of the subject selected. To take part in the study, subjects could send their initial agreement and phone number by using the coupon-answer accompanying the official letter. They could also phone or send an e-mail to the NESCaV research team. Then, an appointment was made with the subject at the nearest appointed medical center involved in the study. Those who refused to participate were no longer contacted and were replaced by subjects presenting the same characteristics. Whenever possible, subjects who did not respond spontaneously after one week were contacted again by phone. The phone number was obtained from the telephone company. At the visit, all participants were duly informed and signed a consent form to take part in the study. Data consisted of information collected from a self-administered questionnaire, a clinical examination, and laboratory tests (blood, urine, and hair tests) for each participant. All participants were informed about their measurements but laboratory test results were sent to their family physician.

### 2.3. Anthropometric and Clinical Measurements

Anthropometric and blood pressure measurements were performed by trained health professionals according to standard recommendations [9, 10]. Before examination, participants were asked to be fasting and to refrain from smoking for at least 8 hours. All patients were weighed on the same calibrated digital scale (SECA 888; precision class I, 93/42/CEE) with subject wearing light clothes without shoes. Subjects stood in the center of the scale, with feet 25 cm apart, looking ahead, and arms hanging freely [9–11]. Height was measured with the same portable stadiometer (SECA 213; precision class I, 93/42/CEE) according to the following protocol: no shoes, light clothes, no hair accessories, standing on guard against the stadiometer, heels together, shoulders in relaxed position, arms hanging freely, and knees straight [9–11]. Subjects were standing with head straight so that the Frankfurt plane was horizontal and eyes were focused forward. During measurement, subjects took a deep breath and stood as straight as possible [9–11]. Body mass index (BMI) was calculated using the standard formula of body weight in kg divided by the squared height in meters (kg/m²) [9–11]. Waist circumference (WC, cm) was measured to the nearest 0.1 cm with the subject in standing position, using a flexible, nondistensible tape (SECA 201) and avoiding pressure exertion on the tissues, at the level midway between 12th rib and the uppermost lateral border of iliac crest during mild expiration with the tape all around the body in horizontal position [9–11]. Blood pressure was measured at least 3 times with a minimum of 1-minute interval between each measurement [11] by using a digital automatic blood pressure monitor (OMRON M6 (HEM-7001-E(V)); precision ±3 mmHg) with an appropriate cuff size adapted to the upper arm perimeter (OMRON CL1). Analyses were based on the mean values of the second and third measurements of systolic (SBP, mmHg) and diastolic blood pressure (DBP, mmHg).

### 2.4. Biochemical Parameters

A blood sample was collected from each participant after an overnight 8-hour fast (including abstaining from smoking). All samples were analyzed at the Laboratory of the University Hospital of Liège (CHU). Fasting plasma glucose (FPG, mg/dL) was determined by the enzymatic hexokinase method (Modular P, Roche). Triglycerides concentration (TG, mg/dL) was measured using the enzymatic glycerol phosphate oxidase/PAP (Modular P, Roche). An enzymatic method with cholesterol oxidase (Modular P, Roche) was used in dosage of total cholesterol (TC, mg/dL). Low-density lipoprotein cholesterol (LDL-C, mg/dL) was assayed by inhibition of other fractions of cholesterol and enzymatic colorimetric method with kit reagents on Roche MODULAR P. High-density cholesterol (HDL-C, mg/dL) was determined by enzymatic colorimetric method with PEG-modified enzymes (Modular P, Roche).

### 2.5. Socioeconomic and Lifestyle Factors

The self-administered questionnaire was used to collect information about demographic and socioeconomic characteristics. In the present study, participants were classified into three age-groups: 20–29, 30–49, and 50–69 years old, respectively. Educational level was categorized into four classes: primary and lower secondary, secondary, bachelor, and university degree. “Subjective” economic level was assessed by asking participants if they had the feeling that their financial resources allowed them to meet their household needs and was coded as “in need” (from very difficult to difficult) or “well off” (from rather easy to very easy). “Objective” economic level was classified as either below or above risk of poverty threshold. According to the Belgian Federal Government website, the poverty threshold is equivalent to 60% of the median disposable income at the individual level. For households, it is calculated by multiplying the threshold of isolated people by the household size. In the present study, subjects with an income below this threshold were considered “below risk of poverty threshold.”

Data regarding lifestyle characteristics, including family and personal diseases history and medication intake, were also collected with the self-administered questionnaire. Family disease history was based on four questions about myocardial infarction, stroke, diabetes, and/or HTA within the participant's family. Personal disease history was based on a more comprehensive list of multiple choices of diseases related to CVD. Nutritional habits were assessed with a validated food frequency questionnaire [12] but not analyzed here.

### 2.6. Definition of CRFs

The study specifically focused on 7 distinct CRFs: hypertension, dyslipidemia, global obesity, abdominal obesity, diabetes, current smoking, and physical inactivity. Participants were classified as having hypertension if they reported taking antihypertensive medications and/or had SBP ≥ 140 mmHg and/or DBP ≥ 90 mmHg [11, 13]. Subjects with dyslipidemia were described as having at least one of the following anomalies, TC ≥ 190 mg/dL, TG ≥ 150 mg/dL, LDL-C ≥ 115 mg/dL, and HDL-C < 40 mg/dL for men and < 46 mg/dL for women, and/or taking lipid lowering medications [11, 14]. Global obesity was defined as BMI ≥ 30.0 kg/m² according to the WHO [11, 15]. Abdominal obesity was assessed as a WC ≥ 102 cm in men and ≥ 88 cm in women [11, 16]. Diabetes was defined when participant reported taking antidiabetic medications and/or had FPG ≥ 126 mg/dL [11, 17]. Current smoking was defined on the basis of self-reported responses (regular and occasional smoker), while past or never smokers were considered nonsmokers. Physical inactivity was defined as the practice of a sport (yes/no) less than once a week (frequency of practice).

### 2.7. Statistical Analysis

Results were expressed as mean ± standard deviation (SD) for normally distributed quantitative variables and as median and interquartile range (P25–P75) for the skewed variables. Frequencies were used to summarize qualitative variables. The estimated prevalence of each CRF was associated with its 95% confidence interval (95% CI). The impact of age and gender on anthropometric, clinical, and biological characteristics was assessed by a multiple linear regression. Logistic regression analysis was applied to assess the effect of age and gender on the prevalence of CRFs. It was also used to test the potential effect of socioeconomic factors on CRF prevalence. To account for the stratified random sampling method, weighted statistical methods were applied. A sampling weight equal to the inverse probability of unit selection was allocated to each subject from the same stratum. This stratum sampling weight was defined as the ratio between the population stratum size and the observed sample stratum size. Results were considered significant at the 5% critical level (*P* < 0.05). All statistical analyses were performed by using the SAS 9.3 survey procedure for complex sampling design (© SAS Institute Inc., Cary, NC, USA).

## 3. Results

### 3.1. Study Subjects

The study enrolled 1017 subjects. Their demographic and socioeconomic characteristics are given in Table 1. There were 511 (50.1%) women and 506 (49.9%) men with a median age of 45.1 years (IQR: 33.4–56.0). A majority of participants (76.8%) reported no financial difficulties.

The anthropometric, clinical, and biological characteristics of the subjects are presented in Table 2 by age category and gender. All anthropometric, clinical, and biological characteristics increased with advancing age (*P* < 0.0001), except for HDL-C which tended to remain stable (*P* = 0.095). A significant gender effect was observed for BMI, WC, SBP, DBP, FPG, HDL-C, LDL-C, and TG where men had higher levels than women, except for HDL-C where levels were lower. TC was not influenced by gender (*P* = 0.16).

### 3.2. Epidemiology of CRFs

By decreasing order of prevalence (Table 3), the most predominant CRF was dyslipidemia 65.7% (95% CI: 62.8–68.6), followed by physical inactivity 55.2% (95% CI: 52.2–58.1), hypertension 31.2% (95% CI: 28.4–34.0), current smoking 25.0% (95% CI: 22.4–27.6), abdominal obesity 23.0% (95% CI: 20.5–25.5), global obesity 18.3% (95% CI: 16.0–20.6), and diabetes 6.5% (95% CI: 5.0–8.0). The prevalence of all CRFs increased with age, except for current smoking where the prevalence decreased with age. Concerning gender, the prevalence of hypertension, dyslipidemia, and current smoking was higher in men than women. Physical inactivity concerned more women than men. Both men and women were equally affected by global and abdominal obesity, as well as diabetes.

### 3.3. Relations between Socioeconomic Factors and CFRs

Table 4 presents the age-, gender-, and district-adjusted odd ratios for all CRFs according to socioeconomic factors. Hypertension, global obesity, abdominal obesity, current smoking, and physical inactivity were more frequent in subjects with low education level. Subjects who consider themselves “in need” were at a higher risk to present hypertension (OR = 1.65; 95% CI: 1.18–2.32), global obesity (OR = 1.91; 95% CI: 1.34–2.74), abdominal obesity (OR = 2.07; 95% CI: 1.46–2.94), current smoking (OR = 1.89; 95% CI: 1.37–2.61), and physical inactivity (OR = 2.40; 95% CI: 1.75–3.31) than people who consider themselves “well off.” People living below risk of poverty threshold were more disposed to present global obesity (OR = 2.0; 95% CI: 1.34–2.98), abdominal obesity (OR = 2.02; 95% CI: 1.36–2.99), current smoking (OR = 1.60; 95% CI: 1.09–2.33), and physical inactivity (OR = 1.86; 95% CI: 1.30–2.66) as compared to people living above risk of poverty threshold.

From a multivariable perspective (Table 5), low level of education was associated with hypertension (*P* = 0.045), global obesity (*P* = 0.031), abdominal obesity (*P* < 0.0001), current smoking (*P* = 0.020), and physical inactivity (*P* = 0.016). Concerning subjective economic level, subjects who consider themselves “in need” were more concerned by hypertension (OR = 1.56; 95% CI: 1.05–2.33), current smoking (OR = 1.57; 95% CI: 1.09–2.28), physical inactivity (OR = 2.04; 95% CI: 1.42–2.93), and abdominal obesity (OR = 1.54; 95% CI: 1.01–2.36) than people living above risk of poverty threshold.

## 4. Discussion

Mortality and morbidity associated with CVD continue to have a major socioeconomic impact in Europe and contribute to significant health inequalities. In Belgium, CVD are the first cause of death. Monitoring the epidemiology of CRFs is important to develop some appropriate recommendations to prevent CVD especially in specific risk groups.

According to the present NESCaV findings, dyslipidemia was the most predominant risk factor for the targeted population of Wallonia, followed by physical inactivity, hypertension, current smoking, abdominal obesity, global obesity, and diabetes. The same observations were made in Grand-Duchy of Luxembourg (GLD) (ORISCAV-LUX) where dyslipidemia concerned 69.9%, hypertension 34.5%, current smoking 22.3%, global obesity 20.9%, and diabetes 4.4% [11]. Except for diabetes and current smoking, the Belgian population evidenced lower prevalence of aforementioned CRFs. This high risk profile correlated well with the mortality rate of CVD, respectively, 36.5% in 2009 in Grand-Duchy of Luxembourg [18] and 31.4% in the same year in Belgium [19]. The comparison with other prevalence studies is difficult because of important methodological differences, sociodemographic profiles differences of the study subjects, and the definition of CRFs that were used. However, the Canadian Health Measures Survey (CHMS) in 2007–2009 indicated 27.1% for hypertension prevalence, 23.0% for current smoking, 22.5% for dyslipidemia, 22.2% for global obesity, and 6.9% for diabetes. The 2005–2008 US National Health and Nutrition Examination Survey (NHANES) reported a prevalence of 40.2% for hypertension, 33.8% for global obesity, 25.9% for dyslipidemia, 24.8% for current smoking, and 11.1% for diabetes [20]. The large difference in the prevalence of dyslipidemia between the results of these two studies and the NESCaV study may probably be explained by a difference in the definition of dyslipidemia.

The prevalence of dyslipidemia, current smoking, and hypertension was significantly higher in men than in women. These observations were consistent with findings of the ORISCAV-LUX survey, except for global obesity. Physical inactivity was more frequent in women than in men. The same observation was made in the 2014 Eurobarometer survey on physical activity [21]. The prevalence of other CRFs was not associated with gender. The absence of gender difference for abdominal obesity is interesting. Typically, men are more affected by abdominal obesity and women are more affected by gynoid obesity [22]. In our study, men and women were equally affected by intra-abdominal adiposity (*P* = 0.35). A recent study in the United States showed that abdominal obesity increased in women between 1999 and 2008 [23]. This is a public health problem which needs to be monitored because abdominal obesity is an indicator of visceral fat accumulation [24] and a predictor of adverse metabolic or cardiovascular outcomes independently of body mass [22].

Physical inactivity, current smoking, global obesity, and abdominal obesity were associated with lower educational and subjective and/or objective economic levels. The prevalence of hypertension was also higher in subjects with low educational level and low subjective economic level. These findings once again indicate that lower socioeconomic groups are associated with unfavorable cardiovascular risk factor profiles. Moreover, they were consistent with socioeconomic inequalities in cardiovascular mortality observed in industrialized countries [6]. Two hypotheses have been proposed to explain the relation between health and socioeconomic status. The first hypothesis, the social causation, suggests that the socioeconomic status influences health and the second hypothesis, the social selection, suggests that poor health limits individual's educational and occupational achievements, leading to lower socioeconomic status in adulthood. Elovainio et al. showed that the relation between socioeconomic status and health is not only unidirectional. In their study, they observed that social selection operates at younger age and social causation contributes to socioeconomic differences in cardiometabolic health in midlife [25]. More attention should be paid to disadvantaged socioeconomic groups for CVD prevention at each stage of life.

The prevalence of current smoking was the same as the prevalence observed in the Belgian Health Interview Survey in 2008 (25%) [26]. In Luxembourg, current smoking concerned 22.3% of the general population [11]. In the European Union, 26% of people aged 15 years and older are current daily smokers [27]. Because smoking cessation reduces the risk of CVD and premature death [28], further efforts should be made to fight smoking in specific risk groups.

In this survey, 44.8% of the subjects reported practicing a sport at least once a week, slightly higher than in EU (41%) [21]. These low prevalence rates show the need to promote physical activity because of its association with favorable effects on most CRFs, namely, abdominal obesity, dyslipidemia, global obesity, insulin sensitivity, and blood pressure [29]. Increasing physical activity is one of WHO recommendations to prevent CVD morbidity and mortality [30].

The main strength of the present study was a fair representativeness of the population of Wallonia aged 20–69 years and the large sample size (*n* = 1017). However, the exclusion of people living in institutions could decrease the prevalence of CRFs. Another strong point was the use of standardized tools and methods for performing physical and laboratory measurements to define CRFs. This should facilitate national and international comparability as recommended by the WHO stepwise approach [31]. Nonetheless, the information about physical activity, smoking, hypertension treatment, dyslipidemia, and diabetes was self-reported and may be subject to social desirability bias or recall bias. The cross-sectional design of our study was another limitation that makes it difficult to establish causal relations.

## 5. Conclusion

The mortality rate related to CVD has decreased in Belgium in the last decades but the present findings demonstrate that the cardiovascular risk profile in Wallonia is still a matter of public health concern. Moreover, they show that subjects with low socioeconomic status, as indicated by educational level and subjective and objective economic level, have an unfavorable cardiovascular risk factors profile. This highlights the importance of adjusting the current public health policies towards those disadvantaged groups to reduce health inequalities.

## Figures and Tables

**Table 1:** Demographic and socioeconomic characteristics of the study subjects (*N* = 1017).

Variable	Category	*n*	Frequency (%)	Median (IQR)
Age (years)		1017		45.1 (33.4–56.0)
	20–29		200 (19.9)	
	30–49		436 (42.4)	
	50–69		381 (37.7)	
Gender		1017		
	Male		506 (49.9)	
	Female		511 (50.1)	
Educational level		1005		
	Primary and lower secondary		268 (26.8)	
	Secondary		274 (27.3)	
	Bachelor		263 (26.0)	
	University		200 (19.9)	
Subjective economic level		1013		
	In need		235 (23.2)	
	Well off		778 (76.8)	
Objective economic level		932		
	Below risk of poverty threshold		169 (18.1)	
	Above risk of poverty threshold		763 (81.9)	

**Table 2:** Anthropometric, clinical, and biological characteristics by age category and gender in the NESCaV sample (*N* = 1017).

Characteristic	Age category (years)			*P* value^a^	*P* value^b^
	20–29	30–49	50–69		
Number of subjects (male/female)	200 (100/100)	436 (218/218)	381 (188/193)		
BMI (kg/m²)					
Male	24.9 ± 0.5	26.5 ± 0.3	28.1 ± 0.3	<0.0001	<0.0001
Female	23.3 ± 0.5	24.8 ± 0.3	26.9 ± 0.4		
WC (cm)					
Male	84.9 ± 1.2	91.0 ± 0.8	98.4 ± 0.9	<0.0001	<0.0001
Female	73.9 ± 1.0	79.3 ± 0.8	85.5 ± 1.0		
SBP (mmHg)					
Male	127.4 ± 1.2	127.7 ± 0.9	134.8 ± 1.2	<0.0001	<0.0001
Female	111.7 ± 1.2	114.0 ± 0.9	126.3 ± 1.2		
DBP (mmHg)					
Male	74.4 ± 0.8	78.9 ± 0.7	82.7 ± 0.7	<0.0001	<0.0001
Female	69.4 ± 1.0	73.8 ± 0.7	77.3 ± 0.8		
FPG (mg/dL)					
Male	85.3 ± 0.6	91.4 ± 1.1	100.4 ± 2.3	<0.0001	<0.0001
Female	79.8 ± 0.6	84.9 ± 0.9	91.5 ± 1.7		
HDL-C (mg/dL)					
Male	57.0 ± 1.4	53.6 ± 0.9	54.5 ± 1.1	0.095	<0.0001
Female	67.6 ± 1.4	66.6 ± 1.1	69.4 ± 1.3		
LDL-C (mg/dL)					
Male	103.5 ± 3.1	126.0 ± 2.1	127.5 ± 2.6	<0.0001	0.002
Female	96.5 ± 2.7	111.4 ± 2.0	130.9 ± 2.5		
TC (mg/dL)					
Male	174.5 ± 3.3	198.9 ± 2.5	203.4 ± 2.9	<0.0001	0.16
Female	179.0 ± 3.1	191.5 ± 2.4	217.9 ± 2.7		
TG (mg/dL)					
Male	88.1 ± 5.7	115.8 ± 5.2	124.9 ± 5.3	<0.0001	<0.0001
Female	88.5 ± 3.8	78.6 ± 2.7	102.6 ± 4.4		

^a^Age effect, ^b^gender effect.

**Table 3:** Prevalence of CRFs by age category and gender in the NESCaV sample (*N* = 1017).

CRFs	Age category (years)			*P* value^a^	*P* value^b^
	20–29	30–49	50–69		
	*n* (%)	*n* (%)	*n* (%)		
Hypertension					
Total	317 (31.2)				
Male	21 (10.4)	58 (13.4)	115 (29.9)	<0.0001	<0.0001
Female	5 (2.5)	34 (7.74)	84 (22.3)		
Dyslipidemia					
Total	653 (65.7)				
Male	38 (19.8)	153 (36.1)	162 (43.1)	<0.0001	<0.0001
Female	34 (17.4)	110 (25.5)	156 (42.0)		
Global obesity					
Total	186 (18.3)				
Male	13 (6.59)	36 (8.34)	48 (12.5)	<0.0001	0.41
Female	9 (4.48)	31 (7.01)	49 (12.9)		
Abdominal obesity					
Total	234 (23.0)				
Male	7 (3.47)	32 (7.36)	71 (18.5)	<0.0001	0.35
Female	10 (4.96)	37 (8.42)	77 (20.3)		
Diabetes					
Total	65 (6.52)				
Male	1 (0.50)	8 (1.89)	27 (7.21)	<0.0001	0.32
Female	2 (0.99)	10 (2.33)	17 (4.53)		
Current smoking					
Total	253 (25.0)				
Male	37 (18.8)	56 (13.2)	45 (11.9)	0.001	0.048
Female	32 (16.0)	47 (10.5)	36 (9.32)		
Physical inactivity					
Total	561 (55.2)				
Male	39 (19.6)	110 (25.4)	114 (29.6)	0.001	0.035
Female	52 (26.0)	127 (28.9)	119 (31.5)		

^a^Age effect, ^b^gender effect.

**Table 4:** Univariate association between socioeconomic factors and CRFs in the NESCaV sample stratified by age, gender, and district (*N* = 1017).

	Hypertension		Dyslipidemia		Global obesity		Abdominal obesity		Diabetes		Current smoking		Physical inactivity	
	OR	*P* value	OR	*P* value	OR	*P* value	OR	*P* value	OR	*P* value	OR	*P* value	OR	*P* value
	(95% CI)		(95% CI)		(95% CI)		(95% CI)		(95% CI)		(95% CI)		(95% CI)	
Educational level		0.018		0.17		0.0003		<0.0001		0.33		0.0003		<0.0001
Primary	1.88		1.30		3.16		5.05		1.87		2.85		2.55	
	(1.19–2.96)		(0.81–2.07)		(1.81–5.51)		(2.88–8.88)		(0.85–4.11)		(1.74–4.67)		(1.72–3.79)	
Secondary	1.86		1.11		2.35		3.66		1.31		2.11		1.77	
	(1.17–2.96)		(0.72–1.70)		(1.33–4.13)		(2.06–6.51)		(0.56–3.07)		(1.30–3.42)		(1.21–2.57)	
Bachelor	1.35		0.83		1.66		2.34		1.14		1.64		1.12	
	(0.83–2.17)		(0.54–1.27)		(0.92–2.99)		(1.29–4.23)		(0.46–2.77)		(1.00–2.70)		(0.77–1.63)	
University	—		—		—		—		—		—		—	

Subjective economic level		0.004		0.36		0.0004		<0.0001		0.66		0.0001		<0.0001
In need	1.65		1.18		1.91		2.07		1.14		1.89		2.40	
	(1.18–2.32)		(0.83–1.66)		(1.34–2.74)		(1.46–2.94)		(0.63–2.06)		(1.37–2.61)		(1.75–3.31)	
Well off	—		—		—		—		—		—		—	

Objective economic level		0.83		0.091		0.0007		0.0005		0.14		0.015		0.0007
Low ^∗^	1.05		1.45		2.00		2.02		1.61		1.60		1.86	
	(0.70–1.57)		(0.94–2.23)		(1.34–2.98)		(1.36–2.99)		(0.85–3.05)		(1.09–2.33)		(1.30–2.66)	
High ^∗^	—		—		—		—		—		—		—	

^∗^Low = below risk of poverty threshold; high = above risk of poverty threshold.

**Table 5:** Multivariable association between socioeconomic factors and CRFs in the NESCaV sample stratified by age, gender, and district (*N* = 1017).

	Hypertension		Dyslipidemia		Global obesity		Abdominal obesity		Diabetes		Current smoking		Physical inactivity	
	OR	*P* value	OR	*P* value	OR	*P* value	OR	*P* value	OR	*P* value	OR	*P* value	OR	*P* value
	(95% CI)		(95% CI)		(95% CI)		(95% CI)		(95% CI)		(95% CI)		(95% CI)	
Educational level		0.045		0.57		0.031		<0.0001		0.45		0.020		0.016
Primary	1.77		1.03		2.41		4.20		1.73		2.28		1.83	
	(1.07–2.94)		(0.61–1.72)		(1.30–4.47)		(2.25–7.85)		(0.77–3.87)		(1.33–3.20)		(1.18–2.83)	
Secondary	1.91		1.10		2.21		3.80		1.13		1.93		1.60	
	(1.17–3.12)		(0.69–1.74)		(1.24–4.00)		(2.06–7.01)		(0.47–2.72)		(1.16–3.33)		(1.08–2.39)	
Bachelor	1.34		0.84		1.66		2.51		1.13		1.59		1.12	
	(0.82–2.20)		(0.54–1.29)		(0.91–3.02)		(1.36–4.67)		(0.47–2.74)		(0.95–2.65)		(0.75–1.66)	
University	—		—		—		—		—		—		—	

Subjective economic level		0.029		0.99		0.082		0.045		0.75		0.017		0.0001
In need	1.56		1.00		1.46		1.54		0.90		1.57		2.04	
	(1.05–2.33)		(0.68–1.48)		(0.95–2.23)		(1.01–2.36)		(0.46–1.76)		(1.09–2.28)		(1.42–2.93)	
Well off	—		—		—		—		—		—		—	

Objective economic level		0.29		0.14		0.12		0.26		0.31		0.71		0.33
Low ^∗^	0.79		1.42		1.44		1.30		1.45		1.08		1.22	
	(0.50–1.23)		(0.89–2.27)		(0.92–2.27)		(0.82–2.05)		(0.71–2.98)		(0.72–1.64)		(0.82–1.82)	
High ^∗^	—		—		—		—		—		—		—	

^∗^Low = below risk of poverty threshold; high = above risk of poverty threshold.
